# Risk of capture is modified by hypoxia and interjurisdictional migration of Lake Whitefish (*Coregonus* *clupeaformis*)

**DOI:** 10.1038/s41598-024-65147-5

**Published:** 2024-08-05

**Authors:** Richard T. Kraus, H. Andrew Cook, Alexis Sakas, Thomas M. MacDougall, Matthew D. Faust, Joseph D. Schmitt, Christopher S. Vandergoot

**Affiliations:** 1 US Geological Survey, Great Lakes Science Center, Huron, OH USA; 2 Ontario Ministry of Natural Resources and Forestry, Wheatley, ON USA; 3 The Nature Conservancy, Dublin, OH USA; 4 Ontario Ministry of Natural Resources and Forestry, Port Dover, ON USA; 5 Ohio Department of Natural Resources, Sandusky, OH USA; 6 Michigan State University, Lansing, MI USA

**Keywords:** Transboundary migration, Great lakes, Acoustic telemetry, Seasonal hypoxia, Fishing exploitation, Animal migration, Freshwater ecology

## Abstract

Interjurisdictional migrations lead to seasonally changing patterns of exploitation risk, emphasizing the importance of spatially explicit approaches to fishery management. Understanding how risk changes along a migration route supports time-area based fishery management, but quantifying risk can be complicated when multiple fishing methods are geographically segregated and when bycatch species are considered. Further, habitat selection in dynamic environments can influence migration behavior, interacting with other management objectives such as water quality and habitat restoration. As a case study, we examined a novel acoustic telemetry data set for Lake Whitefish in Lake Erie, where they migrate through multiple spatial management units that are variably affected by seasonal hypoxia and host a variety of fisheries. Combining telemetry results with fishery catch and water quality monitoring, we demonstrate three exploitation risk scenarios: (i) high risk due to high residency and high catch, (ii) high risk due to high residency in time-areas with moderate catch, and (iii) low risk due to residency in time-areas with low catch. Interestingly, occupation of low risk refugia was increased by the development of hypoxia in adjacent areas. Consequently, fishery management goals to sustainably manage other target species may be directly and indirectly linked to water quality management goals through Lake Whitefish.

## Introduction

Migratory fishes that cross political boundaries can encounter abrupt changes in methods and intensity of fishing. Interjurisdictional (a.k.a., transboundary) migrations complicate stock identification, creating challenges for coordinated management^1–3^. Investigations have often sought to understand how heterogeneity in vital rates among stocks should inform management actions on mixed fisheries^4,5^. Increasingly, there has also been an appreciation that partial migration and contingent behaviors respond to environmental changes, resulting in some modalities with greater exposure to fishing than others^6,7^. A more comprehensive understanding of complex spatial behaviors has been achieved through advances in the application of natural tracers^8,9^ and electronic technologies that telemeter individuals^10^. These data can quantify a seasonal or life-time chronology of time spent within spatial management areas e.g.,^11^, and thus hold promise for evaluating changes in the risk of exploitation. More time spent in one spatial management area versus another could define whether a fish experiences a high risk of exploitation or a refuge such as the situation with marine protected areas^12,13^. For species for which protected areas are not applicable, annual migratory patterns may still translate into cyclical changes in exploitation risk^14^. In combination with spatial information on fishing effort, fish migration behaviors provide a basis to inform spatial management.

The changing risk of exploitation along a migration route equates to time-varying catchability^15^. An example of this situation would be for non-target species that migrate in or out of a spatial management unit where high fishing effort for a target species is occurring. Exploitation of the bycatch species may change dramatically through the migration, based upon the proportion of the stock that entered or left the spatial management unit in question. For the part of the population that occupies the spatial management unit with high fishing effort, risk of bycatch is clearly higher than adjacent spatial units where fishing effort might be lower, but catchability would remain constant as long as fishing methods are consistent. Fishery-independent information on population size in each spatial management unit would be needed to understand such a scenario. Further, this hypothetical does not need to invoke migration modalities or mixed stocks, but rather emphasizes that a stock may be distributed across multiple spatial management units simultaneously, which is often a reality in many exploited fish populations^16,17^. Time-area evaluation of exploitation risk supports mitigation of bycatch and conservation of information-poor or low abundance species by providing an understanding of when and where management action may be most effective^18^.

**Figure 1:**
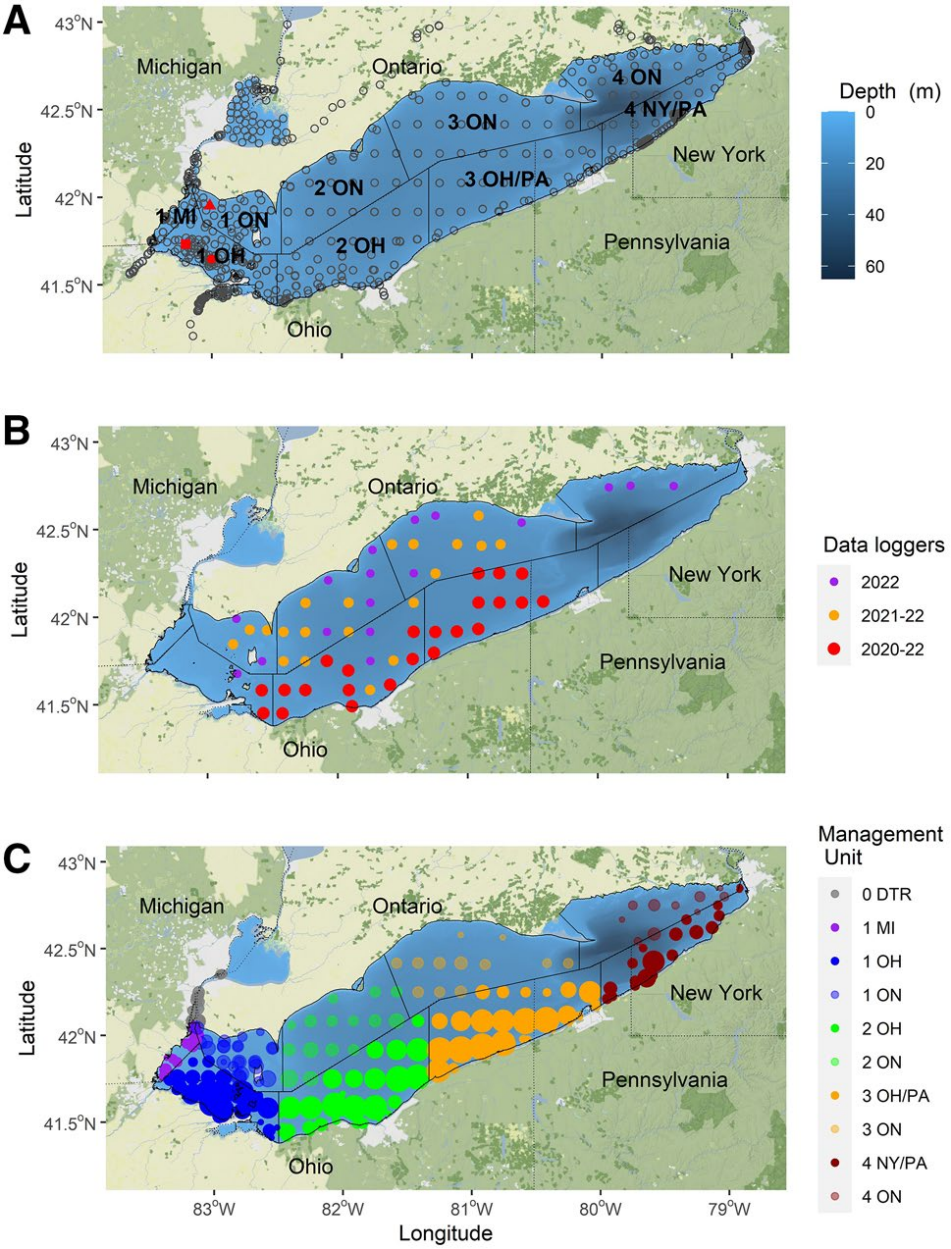
Map of Lake Erie depicting multiple aspects of data analyses in this study: (**A**) spatial management unit (outlined with solid black lines) abbreviations, acoustic telemetry receiver locations (circles), and tagging locations (red symbols: triangle = Colchester Reef, square = Turtle Island Reef, circle = Crib Reef) are plotted on a background of bathymetry; (**B**) locations of receivers equipped with dissolved oxygen data loggers are plotted with colors distinguishing years of deployment; (**C**) acoustic telemetry receiver locations where tagged Lake Whitefish were detected (relative size scales with the number of unique fish, and color shading identifies the jurisdiction and district. Spatial management unit abbreviations are a combination of management districts (0–1–2–3–4, west-to-east), and jurisdictions (*ON* Ontario, *MI* Michigan, *OH* Ohio, *PA* Pennsylvania, *NY* New York. *“0* *DTR”* Detroit River, which is not one of the management areas. Base maps are attributed to the following sources: ©Stadia Maps, ©OpenMapTiles, ©OpenStreetMap.

Exploitation risk can be defined operationally depending on the type of data available, but conceptually, it is the probability of capture, which is difficult to measure directly. Here, we provide an example of estimating changes in exploitation risk throughout cycles of migration by examining Lake Whitefish (*Coregonus* *clupeaformis*) in Lake Erie. Since the implementation of policies in 2014 that discourage harvest, Lake Whitefish are exploited both as bycatch and directly (in a limited commercial trapnet fishery) in an interjurisdictional fishery with five political jurisdictions and nine spatial management units (SMUs). Each jurisdiction has distinct methods and policies for its fisheries, which are heterogeneously distributed across the management units (Fig. 1A). Based on historical catch data, Lake Whitefish are currently in lower abundance and considered data deficient^19–22^. Moreover, successful management of this species is an important component of the Marine Stewardship Council’s sustainability certification (Marine Stewardship Council Certificate Code MSC-F-31199 (F-ACO-0052) [https://fisheries.msc.org/en/fisheries], accessed 1/4/2024) for lucrative percid fisheries, primarily targeting Yellow Perch, *Perca* *flavescens*^23^.

Previous acoustic telemetry research on Lake Whitefish in Lake Erie indicated a bias for a few spatial management units, dependent upon season, and behavioral responsiveness to hypoxic events^24^. In Lake Erie, many Lake Whitefish spawn in the western end of the lake^25^. Lake Whitefish are iteroparous coldwater species that typically spawn during November–December. Those that spawn in the western end of the lake must migrate east to seek out cold hypolimnetic habitats during summer to avoid supra-optimal and lethal temperatures^26,27^ and periodic low oxygen levels^28,29^. We examined a novel acoustic telemetry data set and used fishery-dependent catch data in a case-study approach to address key questions about the annual changes in exploitation risk. Specifically, our objective was to determine if seasonal changes in adult Lake Whitefish distributions during their annual migration resulted in differential residency times in each management unit and whether this translated into spatial–temporal changes in exploitation risk. We hypothesized that exploitation risk would vary seasonally among SMUs, and that this risk may be modified by environmental drivers (e.g., hypoxic events). While the results confirmed expectations, some unexpected results are addressed below in the context of lessons for considering interjurisdictional migrations of fishes.

## Results

Initial sample size of tagged fish was small (n = 99) but allowed characterization of movements and habitat use across Lake Erie; most fish survived for the duration of the expected tag battery life. Due to availability of resources for each participating agency, unequal numbers of fish were tagged at each location (see “Methods”). Some tags were either never detected, were harvested, or were only detected for less than 35 days (n = 29): these were classified as mortalities for this analysis^30^. False detections were removed from the data based upon short detection interval criteria using false detections in the GLATOS R package^31^. The remaining 71 tags produced 614,079 total detections, ranging from 2420 to 33,911 detections per individual fish, recorded on 20 to 116 unique receiver stations per individual fish (Fig. 1C).

**Figure 2:**
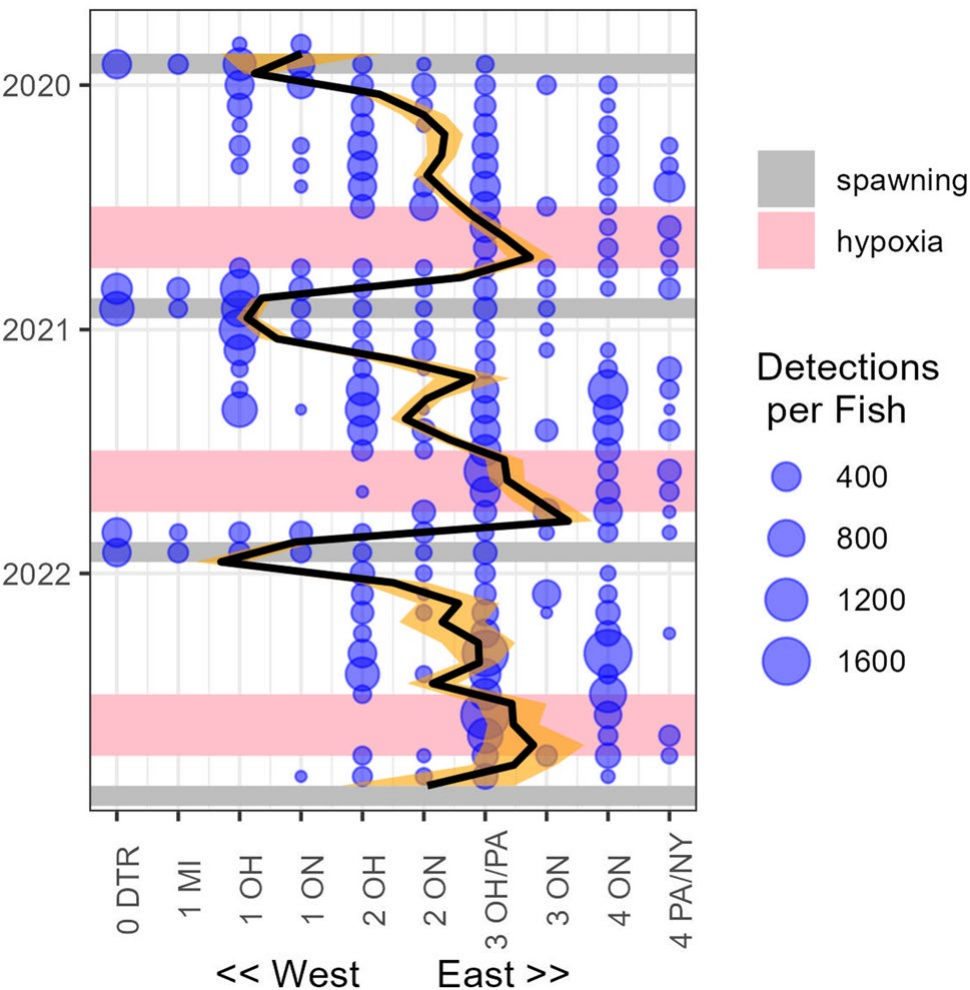
Spatio-temporal summary of detections of tagged Lake Whitefish in Lake Erie. Mean number of detections per tag (blue circles, sizes illustrated in the legend) are summarized for each month (vertical axis) and ordered approximately west-to-east by spatial management unit (horizontal axis). Typical periods of stratification (wide, pink bands) and spawning (thin, gray bands) are plotted for each year. The trend line (black with orange 95% confidence intervals) was estimated tag detections using a linear mixed model. The model was accomplished by assigning numerical values to the spatial management units (SMUs, assigned 1 through 10 as shown in the plot), and predicting SMU as a function of month, weighted by total number of detections (to account for changing numbers of fish at-large), while treating individual fish as random effects. SMU abbreviations are a combination of management districts (0–1–2–3–4, west-to-east), and jurisdictions (*ON* Ontario, *MI* Michigan, *OH* Ohio, *PA* Pennsylvania, *NY* New York. *“0* *DTR”* Detroit River, which is not one of the SMUs.

Movements of fish through the receiver network demonstrated repeatable seasonal patterns with a strong bias toward U.S. jurisdictions (Figs. 1C and 2). Westward movement toward known spawning areas occurred during the end of November and early December, and included detections in Ohio, Ontario, and Michigan waters of District 1 as well as in the Detroit River (here named 0 DTR, Figs. 1C and 2). This movement was followed by a rapid return to Districts 2 and 3 and residence there for spring and early summer (Fig. 2). More detections occurred in Ohio than Ontario during this time (Figs. 1C and 2). A few individuals each year moved farther east toward District 4, but still exhibited an affinity for U.S. waters in Ohio and Pennsylvania (Figs. 1C, and 2). Fish in the central basin (Districts 2 and 3) shifted eastward during months when stratification typically occurs, as indicated both by a lack of detections in District 2 and an increase in detections in Districts 3 and 4 (Fig. 2). After stratification, primarily during late October and November, fish moved westward, assumedly enroute to spawning areas. On this return migration, they exhibited greater use of habitats in Ontario, and less bias toward U.S. waters of Ohio than in other times of the year (Fig. 2).

**Figure 3:**
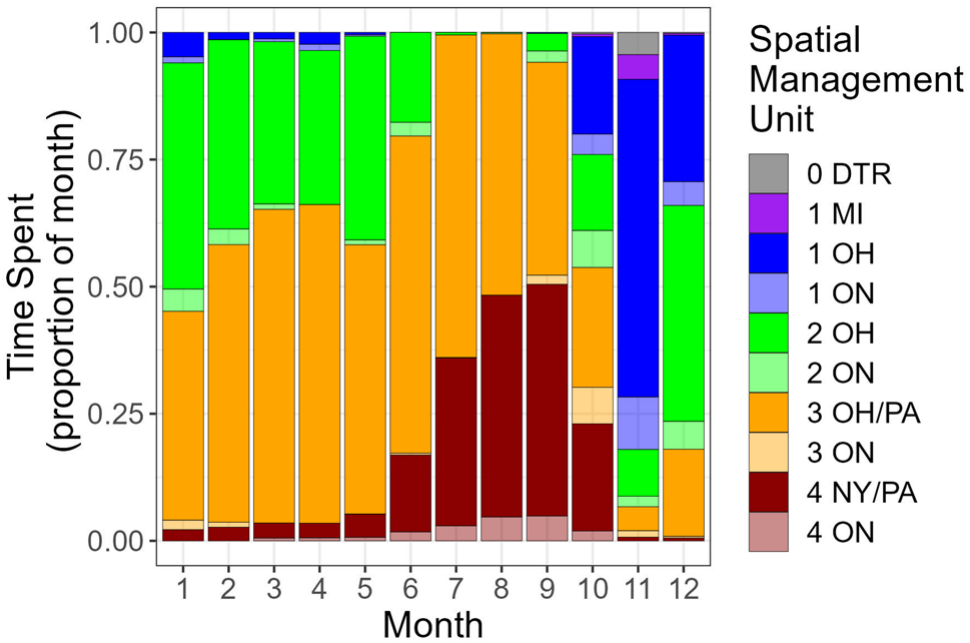
Monthly mean time spent (calculated in days) by tagged Lake Whitefish in spatial management units (SMUs) of Lake Erie from 2019 through 2022. Time was estimated between first and last detections each month in each SMU for individual tags, and time between last and first detections in different SMUs was divided evenly to account for all days of each month. Estimates were averaged across months and fish. Time was scaled to 100% because of the slight variation in length of each month. SMU abbreviations are a combination of management districts (0–1–2–3–4, west-to-east), and jurisdictions (*ON* Ontario, *MI* Michigan, *OH* Ohio, *PA* Pennsylvania, *NY* New York. *“0* *DTR”* Detroit River, which is not one of the management areas.

**Figure 4:**
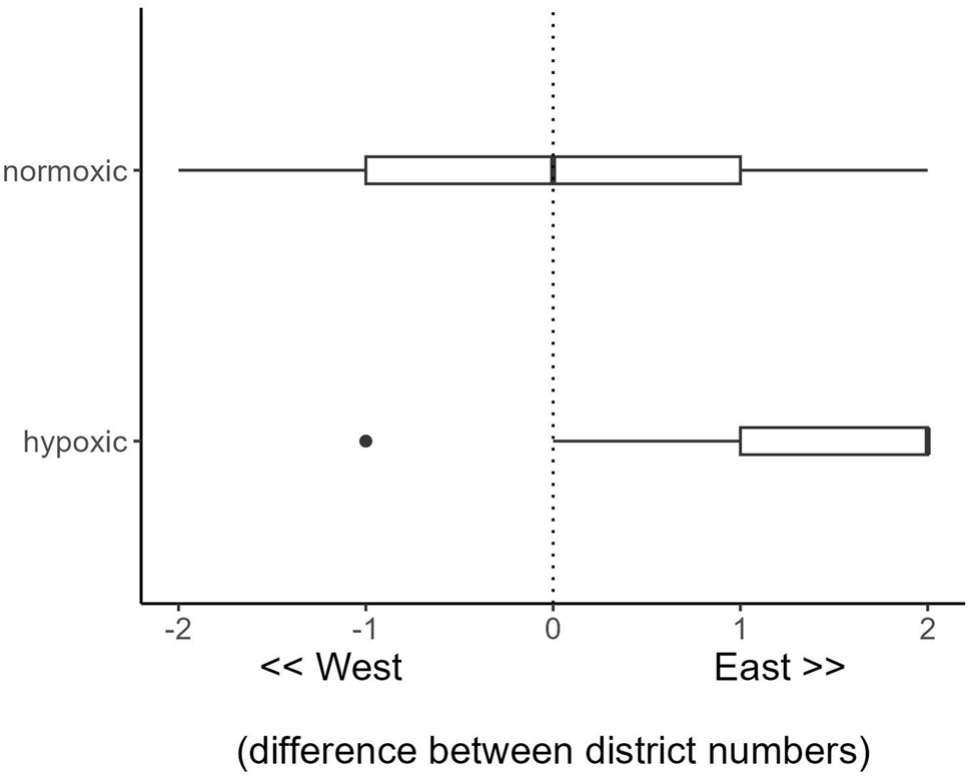
Longitudinal movement of tagged Lake Whitefish between spatial management districts in Lake Erie during stratification in 2021 and 2022. Bottom dissolved oxygen conditions (vertical axis) at the initial and final receivers were estimated from the Lake Erie hypoxia forecast model, defining hypoxia as < 2mg/L. Districts were numbered 1–2–3–4 from west-to-east, and only cases when fish changed spatial management units (SMUs) were considered. Fish that moved latitudinally between SMUs and remained in the same district were assigned a value of zero.

Summarizing time spent in each spatial management unit by month highlighted three distinct modes, which emphasized the movement patterns observed in the detection data (Fig. 2). First, from January through June, Lake Whitefish spent most of the time, > 75% of days each month, in U.S. waters of districts 2 and 3 (Fig. 3). Second, from July through September (stratified months with developing hypoxia), there was a prominent increase in occupation of district 4, peaking at over 50% of days in September (Fig. 3). The near absence of time spent in district 2 during this second mode illustrated the tendency to move east during stratified months. Third, from October through December, all the spatial management units were occupied, peaking in district 1 in Ohio (62% of days) with some use of Michigan waters and the Detroit River in November (Fig. 3). At the district level, the second mode was strongly influenced by hypoxia (Fig. 2). During stratified months, when forecast modeling could be used to estimate mean dissolved oxygen in the hypolimnion, movements were strongly biased eastward when initial conditions were hypoxic, and fish were most frequently displaced eastward by two spatial management units (Fig. 4). By comparison, no longitudinal movement bias could be detected when initial conditions were normoxic (Fig. 4).

While limited to a smaller spatial coverage, data loggers provided direct (as opposed to forecasted) measurements of near-bottom dissolved oxygen values, but results from the integration of tag depths with data loggers produced a more subtle response than we expected. First, when conditions were normoxic, fish tended to reside close to the bottom (Table 1A; mean = 1.7 m > than receiver depth), emphasizing selection of demersal habitats. Several factors may have contributed subterranean tag depths during hypoxic initial conditions (Table 1A; i.e., those appearing greater than the water depth at the receiver location), including (i) normal fluctuations in lake level post-receiver deployment, (ii) detection-at-a-distance when fish were deeper than the receiver location, (iii) accuracy of tag depth sensors (reported to be ± 0.9 m), and various environmental factors^32,33^. The relative influence of these factors could not be quantified with existing data, but their effects were clearly important when conditions were hypoxic. For cases when fish moved between pairs of receivers with data loggers, tag depth increased more than when conditions were hypoxic (mean = 0.3 m), but the difference was small and not significant (Table 1B). Similarly, when fish moved between pairs of receivers, the difference in dissolved oxygen was also small: a slight increase of 0.4 mg/L when conditions were hypoxic and a slight decrease of 0.1 mg/L when initial conditions were normoxic (Table 1C). Despite small changes in average depth or dissolved oxygen between pairs of receivers with successive detections, fish changed location far more frequently when initial conditions were hypoxic (75%) than when they were normoxic (63%; Table 1D).

**Table 1:** Estimates of four metrics from a subset of acoustic telemetry detection data that included only acoustic receivers equipped with near-bottom dissolved oxygen (DO) data loggers. The metrics were estimated with a linear mixed model with dissolved oxygen category as the main effect and tag as a random effect. Hypoxia was defined as DO < 2.0 mg/L, and the difference between DO categories were also estimated. For B) and C), the data were further reduced to pairs of detections on different receivers (indicating movement), with the dissolved oxygen category was defined by initial conditions at the receiver.

Test/initial conditions	Hypoxic	Normoxic	Difference
(A) Difference between tag and receiver depth (m)	**− 0.314 (− 0.99, 0.362)**	**1.697 (1.31, 2.09)**	**2.012 (1.442, 2.581)**
(B) Change in tag depth (m)	0.256 (**− **0.395, 0.552)	0.0001 (**− **0.06, 0.06)	**− **0.256 (**− **0.553, 0.041)
(C) Change in dissolved oxygen (mg/L)	**0.408 (0.204, 0.611)**	**− 0.068 (− 0.11, − 0.03)**	**− 0.476 (− 0.681, − 0.271)**
(D) Proportion of days moved	**0.748 (0.661, 0.835)**	**0.633 (0.588, 0.679)**	**− 0.115 (− 0.196, − 0.034)**

*Least-square type 95% confidence intervals in parentheses. Emboldened values are significantly different than zero.

**Figure 5:**
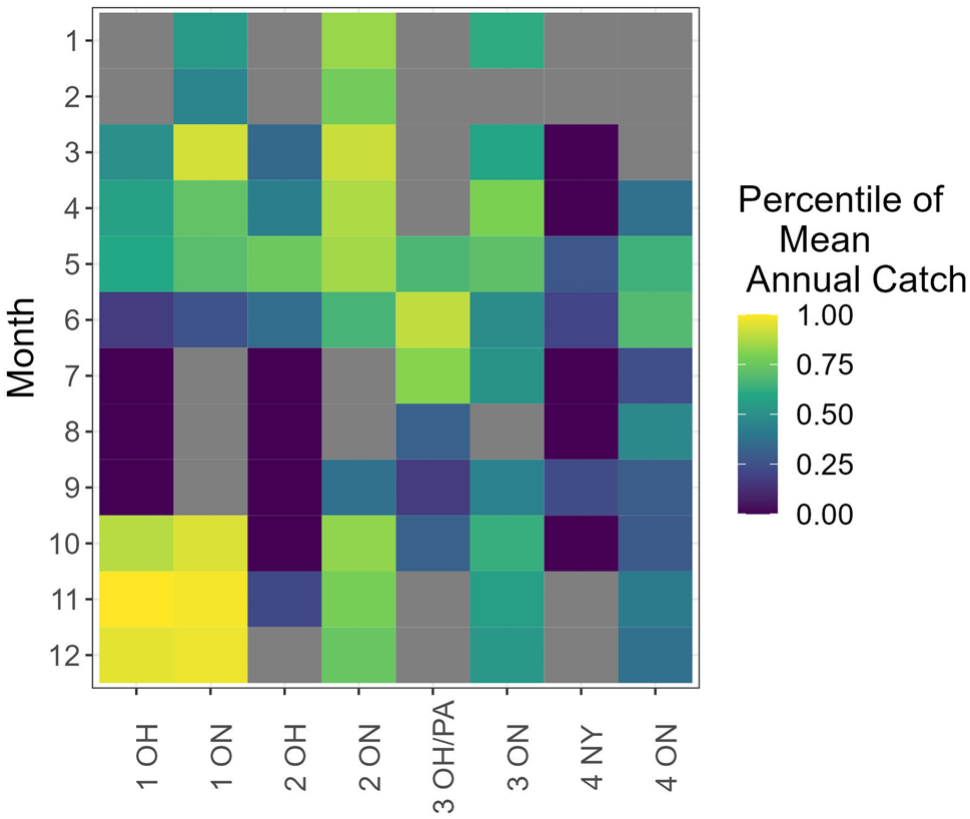
Mean commercial catch (summarized from 2019 through 2022) of Lake Whitefish from demersal fishing gears expressed as a cumulative percentage rank from the entire matrix. The highest levels were observed in district 1 during November as indicated by the color gradient in the key. Zeros were excluded from the calculations and are expressed as gray blocks. SMU abbreviations along the horizontal axis are a combination of management districts (0–1–2–3-4, west-to-east), and jurisdictions (*ON* Ontario, *MI* Michigan, *OH* Ohio, *PA* Pennsylvania, *NY* New York. Not all SMUs are plotted because of the absence of catch in some SMU-month combinations.

**Figure 6:**
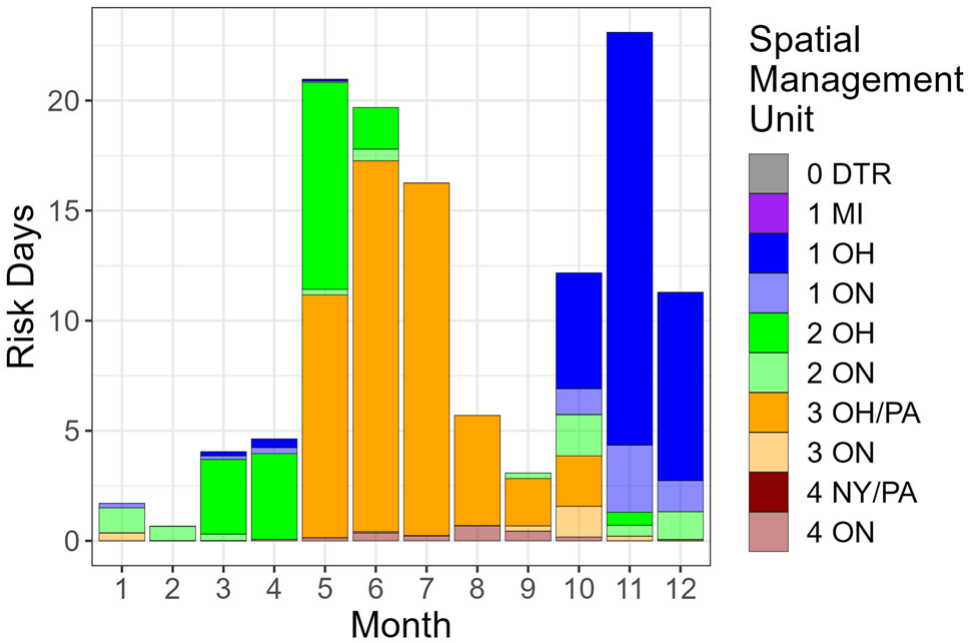
Monthly estimates of risk days for tagged Lake Whitefish at-large in Lake Erie from 2019 through 2022. Risk days are a proxy measurement for probability of capture in the fishery. Risk days were calculated as the product of average monthly time spent in a spatial management unit (SMU) and the rank percentile of reported catch from the entire lake-wide data set (which only included data from the at-large period of tagged fish). SMU abbreviations are a combination of management districts (0–1–2–3-4, west-to-east), and jurisdictions (*ON* Ontario, *MI* Michigan, *OH* Ohio, *PA* Pennsylvania, *NY* New York. *“0* *DTR”* Detroit River, which is not one of the management areas. Not all SMUs are plotted because of the absence of catch in some SMU-month combinations.

Fishery catches of Lake Whitefish are reported annually in a binational report by the Lake Erie Committee’s Cold Water Task Group^34^. The subset of those data, limited to the period of tag detections and limited to demersal gears, illustrated highly variable catch of Lake Whitefish dependent upon month and spatial management unit. The majority of Lake Whitefish were captured during November in Ohio waters of District 1 (Table 2), and this was the result of a small, short-duration (circa 1-month) trap net fishery that targets Lake Whitefish spawning aggregations before winter ice formation limits gear deployment and before policies end commercial fishing on 15 December each year. Smaller but still high overall catches occurred in Ohio in District 1 in October and December. Although incidental, catches in Ontario waters of District 1 were also high, reflecting the concentration of Lake Whitefish in this region during the period surrounding spawning (Table 2). By comparison, incidental catches at other times of the year were much smaller, and primarily occurred during late spring and early summer in districts 2 and 3, both in Ohio and Ontario (Table 2). Calculation of rank percentiles of mean catch across months and spatial management units recapitulated the catch data, emphasizing the highest catches in District 1 in November and to a lesser degree Districts 2 and 3 during spring and early summer (Figs. 5, 6).

**Table 2:** Mean commercial catch (reported in pounds) of Lake Whitefish from demersal fishing gears, 2019 through 2022. Cumulative distributional percentage rank from the entire matrix is shown in parentheses (zeros were excluded from the calculations).

Month/SMU	1 OH	1 ON	2 OH	2 ON	3 OH/PA	3 ON	4 NY/PA	4 ON
January	0	119 (54%)	0	468 (84%)	0	159 (61%)	0	0
February	0	43 (46%)	0	383 (77%)	0	0	0	0
March	56 (50%)	1452 (93%)	12 (34%)	1396 (91%)	0	133 (59%)	0	0
April	128 (57%)	322 (73%)	17 (43%)	753 (87%)	0	452 (80%)	0	14 (37%)
May	147 (60%)	236 (70%)	338 (76%)	565 (86%)	193 (67%)	263 (71%)	5 (27%)	162 (64%)
June	1 (17%)	5 (26%)	14 (36%)	191 (66%)	1261 (90%)	56 (49%)	2 (20%)	199 (69%)
July	0	0	0	0	456 (81%)	80 (51%)	0	4 (24%)
August	0	0	0	0	6 (31%)	0	0	55 (47%)
September	0	0	0	14 (37%)	1 (17%)	21 (44%)	4 (23%)	6 (30%)
October	932 (89%)	2189 (94%)	0	460 (83%)	6 (31%)	160 (63%)	0	6 (29%)
November	31,265 (100%)	6723 (99%)	2 (21%)	417 (79%)	0	124 (56%)	0	14 (41%)
December	2970 (96%)	5826 (97%)	0	328 (74%)	0	81 (53%)	0	14 (37%)

Districts: 1–2–3–4 (west to east).

Jurisdictions: *OH* Ohio, *ON* Ontario, *PA* Pennsylvania, *NY* New York.

The product of mean days per month and rank percentile, a metric we termed risk days, revealed two main results. First, as expected, November was the month with the greatest risk of exploitation (Fig. 6), primarily due to the combination of high proportion of time spent in District 1 (Fig. 4) and high trap net catches in Ohio (Table 2, Fig. 5). Second, the importance of catch quantity was emphasized during September in District 4, where Lake Whitefish spent substantial time in New York waters (Fig. 1C) and catch was very low or absent, resulting in a refuge scenario with a nearly imperceptible quantity of risk days in U.S. jurisdictions (Fig. 6). Also of significance were the months of May through July, which had high overall risk day values that were similar in magnitude to November (Fig. 6). Despite relatively low catch (Table 2), this early spring and summer period of high risk was primarily driven by the substantial time the Lake Whitefish spent in District 3 (and to a lesser degree District 2; Fig. 3).

## Discussion

Lake Whitefish are a migratory species with complex interjurisdictional movements in the Laurentian Great Lakes^5,24,35^. In Lake Erie, acoustic telemetry of Lake Whitefish demonstrated cyclical annual eastward migration from spawning areas in the western end of the lake. Combined with habitat use and fishery-dependent data, our analysis has the potential to support goals of improved management by quantifying periods and spatial management units with high versus low risk of exploitation. Time-area evaluation of exploitation risk from fishing is a growing area of applied fishery science^36–40^. A prominent example is the bycatch of marine turtles, which may be mitigated through knowledge of migration corridors to identify time-area fishing restrictions^18^. The highest risk of Lake Whitefish catch occurred in Ohio’s trap net fishery both during early summer and November, highlighting a potential migration corridor along the southern shoreline of the lake^24^. High affinity for migrating within US waters of Lake Erie also reduced the estimated risk of exploitation by Canadian gill net and trawl fisheries. Further, telemetry data showed that Lake Whitefish were highly demersal, which likely contributed to their responsiveness to seasonal hypoxia in the central basin. Movements of fish during stratified conditions demonstrated active avoidance of hypoxic conditions and revealed a spatiotemporal refuge during late summer and early fall in US jurisdictions of New York and Pennsylvania (despite limited commercial trap net deployment in these areas, as indexed by catch; Table 2).

Although results presented here strongly support a single pattern of habitat use for Lake Whitefish in Lake Erie, other components of this population or other spawning groups in Lake Erie may behave differently. Suspended gill nets (a.k.a., canned and kited in the industry) in Ontario waters accounted for 81% of total Lake Whitefish catch from 2019 to 2022 (Ontario commercial fishery data obtained from Megan Belore, Ontario Ministry of Natural Resources and Forestry, October 2023). This portion of the commercial catch was not utilized to calculate the risk percentile (due to depth telemetry information), but would elevate exploitation risk in Ontario waters relative to Ohio (where suspended nets are not utilized by the fishery). Suspended nets varied in distribution through the water column, but were most often positioned in epilimnetic depths (< 16 m) that our tagged Lake Whitefish inhabited rarely or only partially when bottom depths were shallow such as near bathymetric features (i.e., islands, reefs, or shorelines). Harvest reporting is by license, gear type, effort quantity (i.e., length of net), geographic grid location, net mesh size, district, lake depth, and gear depth. While beyond the scope of this study, more detailed analysis of catch from Ontario commercial gears as a function of gear characteristics would provide information from a broader range of Lake Whitefish body sizes. For example, gear-specific demographic information would provide a test of the hypothesis that suspended gill net catches are primarily comprised of younger, smaller individuals similar to Walleye^41^. Suspended gill net catches could represent subpopulation or demographic components (e.g., contingents) or other populations (e.g., spawning aggregations in other parts of the lake) with migration and habitat selection behaviors that are different and distinct from those represented by tagged fish. Support for multiple distinct populations comes from knowledge of the historical distribution of spawning locations^42^ as well as more contemporary broad-scale sampling documenting the presence of eggs in areas outside of the western basin^43^ and documenting genetic differences^44^. Available data are insufficient to test whether independent populations of Lake Whitefish may exhibit unique habitat selection behaviors with physiological differences that permit occupation of warmer epilimnetic habitats, but ongoing work to examine and characterize habitat based upon temperature and depth may provide future insights.

That environmental conditions can affect fish migration and habitat selection is well established, and there is a growing understanding of how climate change and related anthropogenic disturbances can alter animal distributions^45,46^. In Lake Erie, the severity and extent of hypoxia have increased since the mid-1990s, primarily linked to increased eutrophication via soluble reactive phosphorus loading^47–49^. Efforts to reduce nutrient inputs are aimed at reducing harmful algal blooms and hypoxia^50^, but long-term increasing temperature trends may reduce oxygen solubility, hampering achievement of these goals^51^. Several of our results provide insight on how the current situation or increased hypoxia may interact with the behavior of Lake Whitefish. Specifically, a response to seek out deeper habitats and habitats in the eastern end of the lake indicated (i) that Lake Whitefish may be able to find refugia under a broad range of conditions^52^, and (ii) the response to hypoxia would also tend to reduce exploitation risk by increasing time spent in spatial management units with low fishing effort (eastern management units in Pennsylvania and New York). The hypoxia effect could potentially lead to a situation where reduced fishery catches of Lake Whitefish in the central basin would suggest population decline, while at the same time increasing fishery-independent catches in the eastern end of the lake typically surveyed during August^34^, would indicate a population increase. Thus, directly but through several steps, the objectives of water quality management are linked to stock assessments and fishery management through the impact of hypoxia on fish habitat occupancy. An important aspect of these linkages is how target species (e.g., percids) respond to the same environmental changes and what influence this might have on the distribution of fishing effort and attendant catch of Lake Whitefish.

A stronger avoidance of hypoxia was expected based upon a previous analysis^24^, but methodological differences may help explain the disparity. In the previous study, tags were not equipped with depth sensors and dissolved oxygen values were predicted from the hypoxia forecast model^53^. In the present study, point measurements of dissolved oxygen from data loggers on the receivers could be matched with tag detections. While the forecast model was able to predict dissolved oxygen at every receiver location, the analysis with data loggers had important limitations: (i) loggers were only deployed in the central basin, (ii) only movement between locations with data loggers were included in the fine-scale analysis (i.e., broader movements between sub-basins of Lake Erie were not considered), and (iii) due to the pandemic, only receivers in U.S. waters could be equipped with data loggers in 2020. Thus, sample size of movements for data loggers was limited and is less comparable to the greater contrast in modelled dissolved oxygen conditions among basins as examined in Kraus et al.^24^. Further, as noted in the previous study, one of the areas of greatest forecast model uncertainty was the eastern portion of the central basin of Lake Erie, which is where tagged fish typically spent most of their time during the stratified period. The distribution of hypoxia is often more heterogeneous^54,55^ on smaller spatial scales than the distance at which tags can be detected (ca. 1 km), and deeper areas frequently have higher (normoxic) dissolved oxygen than depths near the edge of the thermocline, especially early in the development of hypoxia^53^. The thermocline depth in Lake Erie (typically around 15 m) is associated with steeper bathymetry^56^. There were many observations of tag depth that were greater than receiver depth (i.e., lake depth), which likely represent cases that are explained by fish occupying deeper adjacent habitats while being detected from a distance. Thus, it would be possible and likely for hypoxia to be recorded at data loggers, while tagged fish were detected in deeper normoxic refugia. One way to test this hypothesis in the future would be through combined use of data loggers and fine-scale positioning with a denser acoustic receiver array^57^.

The scaling of relative catch across gear types or fishing methods, while illustrative for our case example, has important limitations and is unlikely to be appropriately extended to target species in some circumstances. Ideally, standardization of catch rates across gear types^58,59^ would be desirable to be able to quantify equivalent units of effort between gear types so that risk can be evaluated in areas where fishing, but no catch, occurs. Likewise, such alternative metrics would increase understanding how to compare low versus high effort scenarios when no catch occurs. Because fishing effort tends to track the spatial distribution of target species and gear types are associated with distinct catchabilities, standardization of effort is essential for developing unbiased estimates of population size and distribution^60^. When target catch and bycatch distributions or movements are uncorrelated, catchability of bycatch species may be uncorrelated with catch. As explained in the introduction, this is because changes in the underlying distribution of the bycatch species result in a varying level of catchability that may be unrelated to how the fishery operates. Yellow Perch and Lake Whitefish have substantially different habitat requirements and sensitivity to hypoxia^61–64^, supporting the notion of uncorrelated distributions. Otherwise, if only a single gear type is used and effort is constant, then equating catch as a proxy for risk of exploitation is at least conceptually supported for either target or bycatch species. For the short duration of ca. 2 years for tagged Lake Whitefish, there was only small variation in fishing effort^34^, which provides support for our approach. Importantly, the key to utilizing fishery data to proxy exploitation risk is in conjunction with fishery independent information on the spatial distribution of the fish population. Small scale habitat selection, observed with depth sensors in our example, and broad scale sampling, investigated with the GLATOS receiver network, were critical for understanding how (demersal fishing gear), when (early summer and late fall), and where (U.S. waters) Lake Whitefish could be vulnerable to the fishery. Because our approach depends on retrospective data on Lake Whitefish catch, it cannot predict risk days for novel scenarios of varying effort, although this could be addressed through analyses of effort standardization mentioned above.

Time-area evaluation of Lake Whitefish exploitation risk leverages powerful insights from advanced fish tracking technologies, pointing toward numerous applications for sustainable fishery management as well as native species restoration and conservation. Spatial management in Lake Erie is based on international allocation of quotas to defined areas, and is representative of numerous other marine and freshwater migratory fisheries^1,65^. In Lake Erie, ongoing telemetry work aimed at restoration of Lake Sturgeon, *Acipenser* *fulvescens*^66^, and Lake Trout, *Salvelinus* *namaycush*^67^ is developing fishery-independent information that would allow managers to consider multi-species fisheries in the context of diverse management objectives. While these scenarios may be viewed from the perspective of mitigating impacts on rare species or species of conservation concern, our example of Lake Whitefish also illustrates how managers could use a similar analysis for target species to aid fishery stakeholders. As changes to climate alter habitats and ecosystems, fish may change behaviors, impacting traditional methods. Analyses like the Lake Whitefish example, but for target species, could be used to identify alternative methods that both encourage participation in recreational fisheries and identify opportunities for more sustainable commercial fishing. In this regard, most managed fisheries are well-equipped with requisite data, and the fishery-independent investigations of complex spatial life-histories are rapidly advancing to parity.

## Methods

Tagging of Lake Whitefish was conducted during spawning in November of 2019 through an interagency coordinated effort, involving the Ontario Ministry of Natural Resources and Forestry, The Nature Conservancy, the Ohio Department of Natural Resources, and the U.S. Geological Survey. Three spawning areas were targeted in two jurisdictions: Ontario, Canada, and Ohio, USA (Fig. 1A). Fish were obtained from commercial fishers, agency boat electrofishing, or agency-deployed gill nets with consideration of Lake Whitefish health upon retrieval. We focused on this single-year data set because, unlike previous and subsequent tagging efforts, depth-transponding tags were deployed to better understand habitat use. While all tags used the same technology (manufactured by Innovasea, Inc., Nova Scotia, Canada), different tag models were deployed in each jurisdiction (in Ohio near Crib Reef, n = 50, model V13-TP ADST; in Ohio near Turtle Island, n = 15, model V13-TP; and in Ontario near Colchester Reef, n = 34, model V16TP or V16; Fig. 1A). Due to the differences in tag models and associated battery life, the potential track durations varied from 584 days for the ADST tags and 824 days for the V13TP tags deployed in Ohio, to 1825 days for the V16TP and V16 tags deployed in Ontario. Note that “TP” in the tag model indicates the presence of temperature and pressure (depth) sensors. Habitat analyses using these data are part of a separate manuscript, but here we examine depth from the subset of tags as described below. Surgical implantation of the tags in to the body cavity was carried out according to each government agency’s approved protocols, which met applicable legal requirements of the federal Animal Welfare Act (https://www.nal.usda.gov/animal-health-and-welfare/animal-welfare-act) and followed accepted guidelines for animal care^68^ and previous successful studies on Great Lakes fishes^69–71^. Further, the work was carried out in compliance with ARRIVE guidelines (https://arriveguidelines.org). Detections of tagged fish were obtained (on 20th July, 2023) from the Great Lake Acoustic Telemetry Observation System GLATOS^72^, which is a network of autonomous passive acoustic receivers (primarily VR2W-69 acoustic receivers; Innovasea, Inc., Nova Scotia, Canada). In Lake Erie, GLATOS receivers were deployed in a systematic grid sampling design augmented by the addition of nearshore receivers for targeted research on other species (Fig. 1A).

All tagged fish were adults that varied in total body length based upon the supply of fish available at each tagging location. The largest fish were from Crib Reef (mean total length = 612 mm, s.d. = 30.6, n = 50), followed by Colchester (mean total length = 514 mm, s.d. = 53.1, n = 34) and Turtle Island (mean total length = 453 mm, s.d. = 11.7, n = 15). At these adult body sizes, tagged fish were not considered to be vulnerable to predation by avian (e.g., cormorants or gulls) or common piscine predators (e.g., Walleye, *Sander* *vitreus*) that would consume smaller-bodied but closely related species in Lake Erie^73^. Although recent genetic analysis of single nucleotide polymorphisms suggests discrimination of spawning locations might be possible^44^, a high degree of mixing is evident and neutral genetic markers have failed to resolve geographical differences^74^, and fishery managers treat the species as a single stock in Lake Erie. Thus, despite body size differences in tagged fish at each location, all were pooled for analysis and treated as a single group.

To characterize patterns of seasonal migration and understand how tagged fish utilized SMUs, individual detection histories were summarized monthly by sequential first and last detection dates in each SMU. We used SMU boundaries for the Yellow Perch fishery because these multi-jurisdictional SMUs include all areas of Lake Erie Fig. 1A, and see: Ref.^22^. SMUs are a combination of four districts ordered from west to east (dividing the lake approximately by longitude) and five jurisdictions representing four US states (Michigan, Ohio, Pennsylvania, New York) and the province of Ontario, Canada (Fig. 1A). SMU boundaries for the Walleye (*Sander* *vitreus*) fishery are similar, but slightly different from Yellow Perch, and for any specific deployment of fishing gear, target species can vary. When gaps in time occurred between the last detection in one month and first detection in the following month, an even split of the time gap was assigned to each month. This method of addressing gaps in detection ensured that all the days of the month could be accounted for during the entire detection history of each fish, without bias toward a particular sequence order of SMUs. Time spent in each SMU was summed for each month by individual fish. Then time spent in each SMU was averaged across individual fish and years to examine monthly patterns of habitat use, which were examined as proportions graphically and utilized as average number of days for calculations with fishery data.

Lake Erie is a seasonally stratified ecosystem with a thin (1–5 m) hypolimnion in the central basin (mean depth = 18.5 m, containing management districts 2 and 3) that often develops large hypoxic zones during summer and early fall^75,76^. The western basin (management district 1) is shallower (mean depth = 7.4 m) than the central basin and generally lacks a thermocline, whereas the eastern basin (management district 4, mean depth = 24.4) has too great a hypolimnetic volume to develop hypoxia. Due to high nutrient loading on the western end of the lake, a persistent gradient in trophic status ranges from hypereutrophic in management district 1 to oligotrophic in offshore waters of management district 4. The extent and severity of hypoxia in the central basin have changed dramatically through time^77,78^, showing an increasing trend in recent decades^79^. Lake Whitefish are sensitive to low oxygen conditions^27^; thus, a large area of coldwater refuge (ca. 8000 km^2^) may become unsuitable in the central basin, especially during the months of August and September^52^. This phenomenon has been the subject of a hydrographic modeling effort to forecast hypoxia^53^. To understand how tagged Lake Whitefish responded to hypoxia, the forecast model was used to estimate daily mean dissolved oxygen in the bottom layer (varied with lake depth, but typically was 1 to 2 m thick in the central basin) for each spatial management unit during stratified months. Similar to Kraus et al.^24^, occurrences of movement between management units were extracted from the summarized telemetry data and classified as either hypoxic (< 2 mg/L) or normoxic based upon the initial conditions of the management unit. Movements between spatial management units were classified by longitudinal and latitudinal transitions, which were censored to a duration of less than 21 days. Longer durations were considered less informative for understanding responses to hypoxia, and this time window represented simulated maximum time between successive detections^80^. After initial data explorations, longitudinal transitions were evaluated by examining the rank number difference between the end points of each Lake Whitefish movement between management units under each scenario (hypoxic and normoxic). For example, movement east by one SMU would have a value of 1, whereas movement west would have a value of − 1. Latitudinal movements between SMUs in the same district were coded as zero, and distributions of values were compared by quantiles.

Lake Whitefish are characterized as demersal species, but to better define their habitat use in relation to different fishing methods in Lake Erie, we examined the depth information transmitted by the tags. This involved extracting the subset of detections with tag depth and matching these with water depth information from receiver stations. Further, selected receivers each year in the central basin were equipped with data loggers (model: miniDOT, by PME, Inc., California, USA) that recorded temperature and dissolved oxygen in the bottom 1m of the water column (Fig. 1B). These data loggers were deployed from early summer through fall to capture dynamics during the stratified season. Receiver coverage was continuous but spatially limited for short periods due to impacts of the COVID-19 pandemic on fieldwork. For example, while visiting a station, in most cases receivers could be retrieved and deployed on the same day, but in 2020 due to the pandemic, receivers in Ontario waters were retrieved in early June and redeployed in early August, creating a ca. 45-day gap in coverage. Similarly, the pandemic impacted full deployment of receivers that were equipped with data loggers. In 2020, data logger stations were limited to Ohio waters (n = 25 stations, 13 May to 22 October, Fig. 1B), but in 2021 there was broader coverage in both Ohio and Ontario (n = 44 stations, 10 May to 27 October). In 2022, data logger coverage was expanded further (n = 58 stations, 20 June to 3 November). To complement the data loggers, hypoxia forecast model data were obtained by request from NOAA Great Lakes Environmental Research Lab (https://www.glerl.noaa.gov/res/HABs_and_Hypoxia/hypoxiaWarningSystem.html), and spanned typical periods of stratification (June through October) in each year^53^.

Data logger data were time-matched with telemetry detections to categorize detections as either hypoxic or normoxic. Several comparisons were constructed using the tag and data-logger matched observations. First, to test hypothesis of demersal behavior in Lake Whitefish, the difference between tag and receiver depth was compared under observed hypoxic and normoxic conditions. To understand how hypoxia may have influenced movements, we examined the subset of instances when tags were sequentially detected on a pair of different receivers when water quality data loggers were recording. The change in tag depth and difference in dissolved oxygen recorded by the data loggers was compared between hypoxic and normoxic conditions at the initial receiver. Further, during the period when data loggers were deployed, we compared the proportion of instances of movement versus sequential detections on the same receiver (indicating non-movement) under initial conditions of hypoxia or normoxia. We used a mixed effects model with a Gaussian distribution comparing hypoxic and normoxic categories, while accounting for correlated measurements within individual tags as random effects^81^. Parameter estimates and effect sizes were compared via 95% confidence intervals of least-squared estimates with Kenward–Rogers degrees of freedom^82^.

A challenging aspect of interjurisdictional fisheries is the heterogeneity of methods and effort epitomized by Lake Erie. For example, in U.S. waters commercial gill nets are banned and the primary fishing methods are commercial trap nets and recreational angling. By comparison in Canadian waters, commercial gill nets account for most of the harvest, and these are be deployed either on the bottom or suspended from the surface by floats, dependent upon time of year, target species, and region of the lake. Since 2014, Lake Whitefish have been captured incidentally when other species are targeted^34^. One exception to the incidental capture is the Ohio trap net fishery in the western basin, where they are targeted during the spawn period from October to December. Thus, the vulnerability of Lake Whitefish to a fishery could change substantially with the timing of movement among spatial management units. Characterization of Lake Whitefish habitat use was necessary to identify which fishery data to use in the analysis of fishing risk. Initial results confirmed demersal behavior; therefore, catch data from demersal gears was obtained from each jurisdiction for the period of tag deployments in this study. These gears were trap nets, which primarily targeted Yellow Perch in US waters, and bottom gill nets and demersal trawls, which respectively targeted Yellow Perch and Rainbow Smelt, *Osmerus* *mordax*, in Canadian waters. Because of the disparate gears and lack of comparative or standardization studies on effort^83^, we made the simple assumption that the quantity of Lake Whitefish catch was a proxy for risk of exploitation. Accordingly, catch in each spatial management unit was summed for each month and averaged across years. Data (reported in Imperial pounds) were truncated to the period of tag deployment (2019 through 2022) and cumulative rank percentile was calculated for each month-spatial management unit combination. This percentile was treated as a proxy measurement of relative risk of capture. The percentile was then used to calculate a metric we termed *risk* *days*, which was the product of time spent (in days, estimated from tagging) and risk of capture (percentile value). Risk days were then examined graphically to identify time-areas with high and low values. This descriptive approach provided a proof-of-concept which we evaluated considering the need for subsequent comparisons of interjurisdictional fisheries.

## Data Availability

Detection^84^and logger data^85^ are publicly available. The hypoxia forecast model results are also publicly available (Lake Erie Hypoxia Forecast: NOAA Great Lakes Environmental Research Laboratory—Ann Arbor, MI, USA). Catch data are reported on the Great Lakes Fishery Commission website [Great Lakes Fishery Commission—Lake Erie Committee (glfc.org)] and are freely available upon request from state and provincial management agencies.
